# Very high sensitivity of a rapid influenza diagnostic test in adults and elderly individuals within 48 hours of the onset of illness

**DOI:** 10.1371/journal.pone.0231217

**Published:** 2020-05-06

**Authors:** Yuki Seki, Yukio Oda, Norio Sugaya

**Affiliations:** 1 Department of Internal Medicine, Keiyu Hospital, Yokohama, Japan; 2 Department of Clinical Laboratory, Keiyu Hospital, Yokohama, Japan; 3 Department of Pediatrics, Keiyu Hospital, Yokohama, Japan; University of California San Francisco, UNITED STATES

## Abstract

During influenza epidemics, Japanese clinicians routinely perform rapid influenza diagnostic tests (RIDTs) in the examination of patients who have an influenza-like illness, and patients with positive test results, including otherwise healthy individuals, are treated with anti-influenza drugs. However, it was recently reported that the sensitivity of RIDTs was extremely low in adult patients. We examined the sensitivity and specificity of an RIDT that is widely used in Japan, ImunoAce Flu (TAUNS, Shizuoka, Japan), in comparison to reverse transcriptase polymerase chain reaction (RT-PCR). The sensitivity and specificity of the ImunoAce Flu test were 97.1% (95%CI: 93.8–98.9) and 89.2% (95%CI: 84.1–93.1), respectively. The ImunoAce Flu test is designed to not only detect influenza A or B, but also to detect H1N1pdm09 with the use of an additional test kit (Linjudge FluA/pdm). Its sensitivity and specificity for A/H1N1pdm09 were 97.6% (95%CI: 87.4–99.9) and 92.6% (95%CI: 82.1–97.9), respectively. Thus, by consecutively testing patients with the ImunoAce Flu test followed by the Linjudge FluA/pdm test, we are able to diagnose whether a patient has A/H1N1pdm09 or A/H3N2 infection within a short time. The reliability of rapid test results seems to be much higher in Japan than in other countries, because approximately 90% of influenza patients are tested and treated within 48 hours after the onset of illness, when the influenza viral load in the upper respiratory tract is high. From the Japanese experience, RIDTs are sufficiently sensitive and highly useful, if patients are tested within 48 hours after the onset of illness.

## Introduction

In Japan, more than 20 rapid influenza diagnostic tests (RIDTs) are marketed. These are considered core tools for determining whether to start treatment with anti-influenza drugs [1]. During influenza epidemics, Japanese clinicians routinely use RIDTs in the examination of patients with influenza-like illness (ILI), and patients with positive test results, including otherwise healthy individuals, are treated with anti-influenza drugs [2]. In Japan, approximately 20–40 million RIDT kits are used every season [3], which costs approximately 200–400 million US dollars per year.

A total of 4 neuraminidase inhibitors (NAIs) are currently used in hospitals and clinics in Japan. These include oseltamivir, zanamivir, the inhaled drug, laninamivir, and the intravenous drug, peramivir. Moreover, a new RNA polymerase inhibitor, baloxavir marboxil, was approved in 2018, and was widely used in the 2018–19 season [4]. It was reported that over 5 million people were treated with baloxavir in Japan.

Even though over 20 million cases of infection were reported in Japan during the 2009 H1N1pdm pandemic, only 198 deaths were reported nationwide with no deaths of pregnant women [5]. The low mortality rate was attributable to the universal implementation of early treatment with NAIs based on universal testing with RIDTs [1].

The diagnosis of influenza based on clinical symptoms alone is difficult. In the US, antiviral treatment was infrequently prescribed for outpatients with influenza for whom therapy would have been most beneficial [6]. The potential benefits of a rapid and accurate diagnosis of influenza infection include prompt initiation of antiviral therapy [7], fewer ancillary diagnostic tests, fewer hospitalizations, prompt initiation of hospital infection control measures, and less unnecessary antibiotic therapy [8].

It was recently reported, based on a meta-analysis, that the sensitivity of RIDTs, antigen detection tests based on immunochromatography, was as low as 42.6% for influenza A and 33.2% for influenza B in adult patients [9], although the specificity was reported to be over 99%. Another recent systematic review of RIDTs showed similar results [10], reporting that the sensitivity and specificity for influenza A+B in adults were 34.1% (95%CI: 14.0 to 54.1) and 99.2% (95%CI:98.2 to 100), respectively.

However, there was a serious problem in these reports, as they did not report the timing of sample collection for the RIDTs. The sensitivity of RIDTs is dependent on the viral load in the upper respiratory tract, and the viral titers of patients with influenza A virus infection in the upper respiratory tract peak during the first 1–2 days after the onset of influenza infection, and decline to undetectable levels within a week [11]. The WHO Agenda for Public Health noted that the reliability of rapid tests in Japan seems to be higher than that in other countries, possibly because most patients are tested within 48 hours of the onset of illness, when influenza viral load in the upper respiratory tract is high [1].

The difference in clinical manifestations between A/H1N1pdm09 and A/H3N2 is very important in the clinical setting. For example, in young adults with H1N1pdm09, severe viral pneumonia sometimes develops as a complication [12], while elderly patients with A/H3N2 often develop bacterial pneumonia. Thus, it is highly beneficial for clinicians to distinguish between influenza A subtypes when they considering the treatment and prognosis of influenza A patients.

The purpose of this study was to examine the sensitivity and specificity of an RIDT that is widely used in Japan (ImunoAce Flu, TAUNS, Shizuoka, Japan), and to demonstrate its usefulness in the outpatient department of our hospital. ImunoAce Flu was reported to show high sensitivity and specificity in an *in vitro* study using viral cultures [13]. We compared its performance to the results of reverse transcriptase polymerase chain reaction (RT-PCR). ImunoAce Flu is designed to not only detect influenza A or B, but to also detect H1N1pdm09 with the use of an additional kit (Linjudge FluA/pdm; TAUNS, Shizuoka, Japan, for research use only). Thus, we also assessed the sensitivity and specificity of Linjudge FluA/pdm.

## Methods

### Clinical specimens

A total of 457 nasopharygeal swab specimens were collected from adults with ILI (upper respiratory symptoms and/or fever) at the outpatient department of Keiyu Hospital, Yokohama, Japan, in the 2018/2019 influenza season (December 2018 to March 2019). The study protocol was approved by the ethics committee of Keiyu Hospital (No.H27-24 in 2017). All subjects provided their informed consent to participate in our study. Nasopharyngeal swab specimens were tested with an ImunoAce Flu test kit.

### ImunoAce Flu and Linjudge FluA/pdm

**Fig 1:**
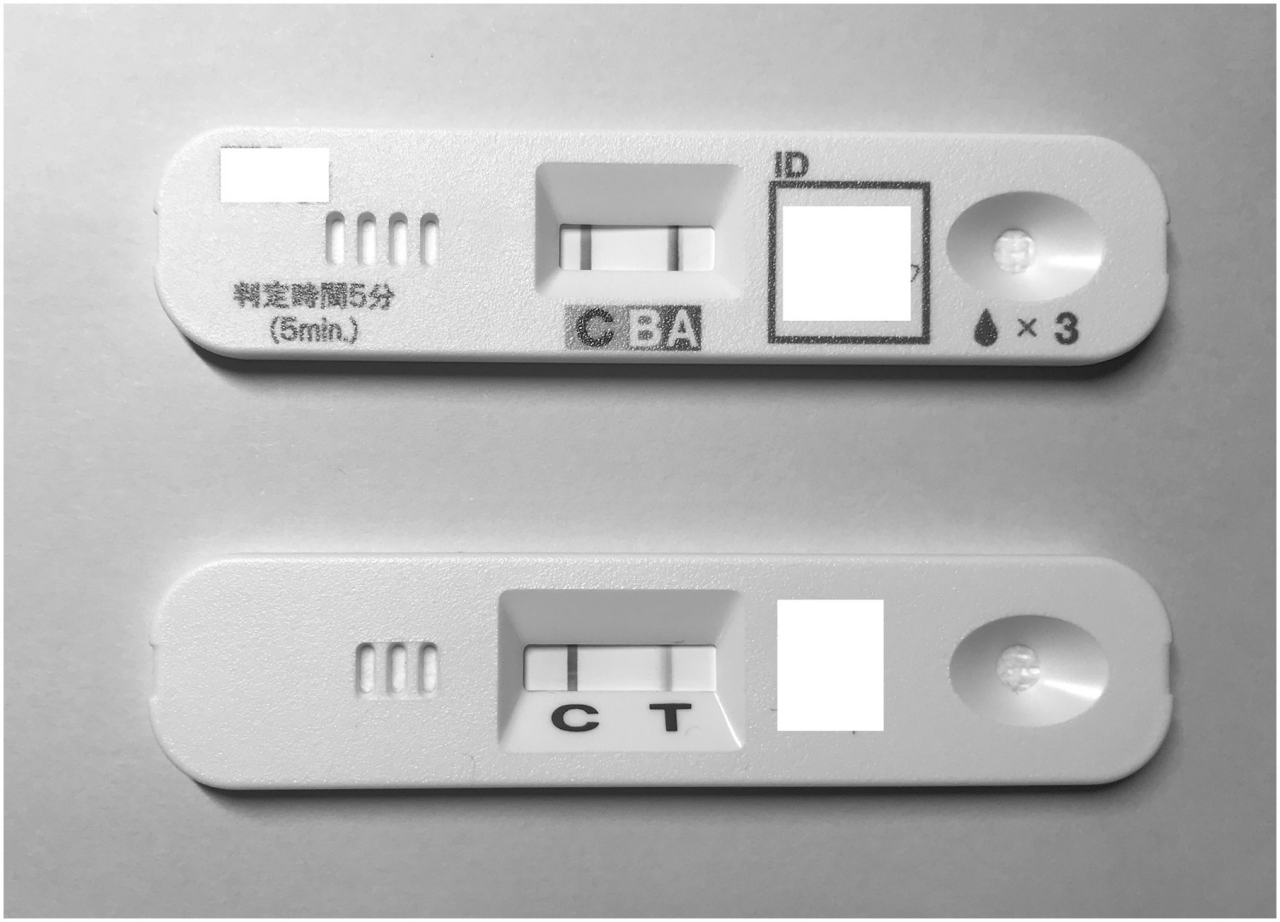
The patient was diagnosed with influenza A/H1N1pdm09 infection because she was found to be positive for influenza A by ImunoAce Flu (upper) and Linjudge FluA/pdm (lower).

**Fig 2:**
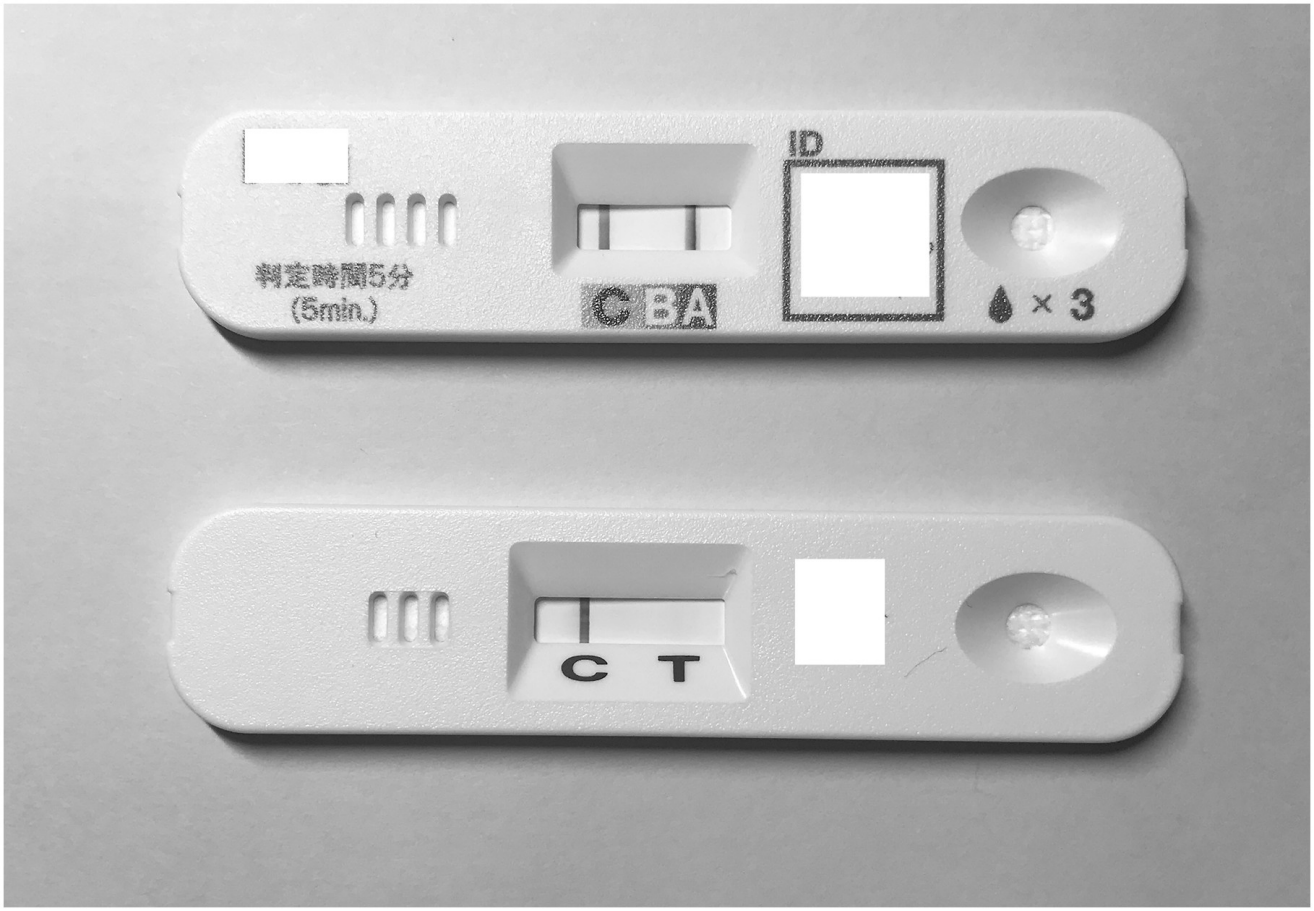
The patient was diagnosed with influenza A/H3N2 infection because he was positive for influenza A by ImunoAce Flu (upper) but negative by Linjudge FluA/pdm (lower).

Samples collected by nasopharyngeal swabs were first tested by ImunoAce Flu [14], and the results were shown in 5 minutes. If patients were positive for influenza A, then the samples were further tested by Linjudge FluA/pdm [15]. This is an RIDT that can only detect A/H1N1pdm09, using a monoclonal antibody that is reactive against nucleoprotein (NP) of influenza A/H1N1pdm09, but not NP of A/H3N2 or seasonal A/H1N1. If a patient was found to be positive for influenza A by ImunoAce Flu and the Linjudge FluA/pdm test yielded a positive result, the patient was diagnosed with A/H1N1pdm09 infection (Fig 1). On the other hand, if the patient was found to be positive for influenza A by ImunoAce Flu, but the Linjudge FluA/pdm test yielded a negative result, the patient was diagnosed with influenza A other than A /H1N1pdm09 (*i*.*e*., A/H3N2 infection) (Fig 2). However, there is a possibility that the patient may have another kind of influenza A infection such as bird influenza, because ImunoAce Flu could detect A/H5N1 and A/H7N9 [13]. These tests were performed in the laboratory department of our hospital.

### RT-PCR

The remaining samples were stored in a refrigerator in the laboratory department of Keiyu Hospital at -20°C. After the epidemic ceased in April 2019, they were sent to the laboratory of TAUNS (Shizuoka, Japan) and tested by RT-PCR. The laboratory that performed RT-PCR testing was not informed of the results of ImunoAce Flu and Linjudge FluA/pdm tests performed in Keiyu Hospital. Keiyu Hospital was informed of the RT-PCR results in June 2019. The results of the ImunoAce Flu and Linjudge FluA/pdm tests performed in Keiyu Hospital were then compared with the RT-PCR results.

RT-PCR samples were extracted using a QIAamp Viral RNA Mini Kit (QIAGEN, Hilden, Germany) from 140 μL of remaining samples after performing the ImuoAce Flu test. Influenza A and B virus detection and the identification of influenza A/H1N1pdm09 and A/H3N2 subtypes was performed by real-time RT-PCR. Reverse transcription and amplification were performed using a One Step RT-PCR Kit Ver.2 (Takara Bio Inc., Shiga, Japan) and real-time RT-PCR was performed using a CFX96 Real-Time System (Bio-Rad Laboratories, Inc., California, USA). Testing was performed according to the Manual for the Diagnosis of Influenza Virus, Version 4 by the National Institute of Infectious Diseases [16].

### Statistical analysis

EZR (Saitama Medical Center, Jichi Medical University, Saitama, Japan), which is a graphical user interface for the R software program (the R Foundation for Statistical Computing, Vienna, Austria) was used to perform the statistical analyses. P values of <0.05 were considered to indicate statistical significance.

## Results

In 2018–19 influenza season in Japan, both influenza A subtypes (A/H1N1pdm09 and A/H3N2) were widely circulated [17]. The scale of the epidemic was the largest since the year 2000, resulting in more than 12 million cases of influenza A virus infection. In contrast, the influenza B epidemic was very small, and mostly involved the Victoria lineage. Thus, influenza B cases were excluded from our analysis.

A total of 457 nasopharygeal swab specimens were collected. Forty-seven specimens were excluded from the analysis for the following reasons: pediatric patient (n = 32), onset of ILI unknown (n = 7), and influenza B virus infection (n = 8). A total of 410 patients were eligible for inclusion in the analysis (male, n = 171; female, n = 239).

The median age of the patients in the study population was 57 years (inter-quartile-range [IQR]: 36–78 years); 42.2% of the patients (173/410) were ≥65 years of age, and the median age of the elderly patients was 80.0 years of age (IQR: 71–87 years).

Most patients (89.8%: 368/410) visited the outpatient department of our hospital within 48 hours after the onset of illness and received an RIDT. Sixty percent (247/410) of the patients visited our hospital within 24 hours after the onset of illness, and received an RIDT.

The overall performance of ImunoAce Flu in comparison with RT-PCR is shown in Table 1. ImunoAce Flu showed high sensitivity (97.1%) in the detection of influenza A virus (95%CI: 93.8–98.9), but relatively low specificity (89.2%; 95%CI: 84.1–93.1). When the subjects were limited to patients tested within 48 hours after the onset of illness, the sensitivity and specificity were 97.9% (95%CI: 94.7–99.4) and 90.4% (95%CI: 85.1–94.3), respectively (Table 2).

**Table 1:** Sensitivity and specificity of ImunoAce Flu according to age group.

	TP^a^	FP^b^	TN^c^	FN^d^	Number of Patients	% Sensitivity	95%CI	% Specificity	95%CI	PPV^e^	95%CI	NPV^f^	95%CI
Total	201	22	181	6	410	97.1	93.8–98.9	89.2	84.1–93.1	90.1	85.4–93.7	96.8	93.1–98.8
<65years	117	16	101	3	237	97.5	92.9–99.5	86.3	78.7–92	88	81.2–93	97.1	91.8–99.4
≥65years	84	6	80	3	173	96.6	90.3–99.3	93	85.4–97.4	93.3	86.1–97.5	96.4	89.8–99.2

^a,^ true positive;

^b,^ false positive;

^c,^ true negative;

^d,^ false negative;

^e,^ positive predictive value;

^f,^ negative predictive value.

**Table 2:** Accumulated sensitivity and specificity of ImunoAce Flu.

Hours after onset	TP^a^	FP^b^	TN^c^	FN^d^	Number of Patients	% Sensitivity	95%CI	% Specificity	95%CI	PPV^e^	95%CI	NPV^f^	95%CI
≦12	36	1	45	0	82	100	85.8–100	97.8	88.5–99.9	97.3	85.8–99.9	100	88.5–100
≦24	158	15	152	4	329	97.5	93.8–99.3	91	85.6–94.9	91.3	86.1–95.1	97.4	93.6–99.3
≦48	186	17	161	4	368	97.9	94.7–99.4	90.4	85.1–94.3	91.6	86.9–95	97.6	93.9–99.3
Total (>48)	201	22	181	6	410	97.1	93.8–98.9	89.2	84.1–93.1	90.1	85.4–93.7	96.8	93.1–98.8

^a,^ true positive;

^b,^ false positive;

^c,^ true negative;

^d,^ false negative;

^e,^ positive predictive value;

^f,^ negative predictive value.

Among elderly patients of ≥65 years of age, the sensitivity and specificity of ImunoAce Flu for influenza A virus were 96.6% (95%CI: 90.3–99.3) and 93% (95%CI: 85.4–97.4), respectively. In contrast, in adults of <65 years of age, the sensitivity and specificity were 97.5% (95%CI: 92.9–99.5) and 86.3% (95%CI: 78.7–92.0), respectively (Table 1). However, there was no significant difference in specificity between the ≥65 years and <65 years age groups (p = 0.757).

Table 2 showed the accumulated sensitivity and specificity of ImunoAce Flu from within 12 hours after the onset of illness to >48 hours after the onset of illness. Within 48 hours after the onset of illness, the sensitivity and specificity were 97.9% (95%CI: 94.7–99.4) and 90.4% (95%CI: 85.1–94.3), respectively (n = 368). At more than 48 hours after the onset of illness, the sensitivity of the RIDT did not decrease, remaining at 97.1%; however, there was a slight decrease in specificity, which fell to 89.3%.

Within 12 hours after the onset of illness, the sensitivity and specificity were 100% and 97.8%, respectively (n = 82). Although the sensitivity did not decrease within 48 hours, the specificity gradually decreased with time, as follows: within 12 hours, 97.8%; 13–24 hours, 88.8%; 25–48 hours, 81.8% (Table 3**)**. After 48 hours, both the sensitivity and specificity decreased. However, there was no statistically significant difference in the sensitivity and specificity between the patients tested within 12 hours after the onset of illness and those tested at more than 48 hours after the onset of illness.

**Table 3:** Sensitivity and specificity of ImunoAce Flu according to time after the onset of illness.

Hours after onset	TP^a^	FP^b^	TN^c^	FN^d^	Number of Patients	% Sensitivity	95%CI	% Specificity	95%CI	PPV^e^	95%CI	NPV^f^	95%CI
≦12	36	1	45	0	82	100	85.8–100	97.8	88.5–99.9	97.3	85.8–99.9	100	88.5–100
13–24	122	14	107	4	247	96.8	92.1–99.1	88.4	81.3–93.5	89.7	83.3–94.3	96.4	91.0–99.0
25–48	28	2	9	0	39	100	82.2–100	81.8	48.2–97.7	93.3	77.9–99.2	100	55.5–100
49-	15	5	20	2	42	88.2	63.6–98.5	80	59.3–93.2	75	91.3	90.9	70.8–98.9
Total	201	22	181	6	410	97.1	93.8–98.9	89.2	84.1–93.1	90.1	85.4–93.7	96.8	93.1–98.8

^a,^ true positive;

^b,^ false positive;

^c,^ true negative;

^d,^ false negative;

^e,^ positive predictive value;

^f,^ negative predictive value.

We additionally performed the Linjudge FluA/pdm test for patients who were found to be positive for influenza A by ImunoAce Flu (n = 96) (Table 4). Six of the 96 cases that were found to be positive for influenza A by ImunoAce Flu were found to be false-positives by RT-PCR. Thus, these were excluded from the analysis. Among the patients in whom influenza A positivity was confirmed by RT-PCR (n = 90), 42 patients were found to be positive for H1N1pdm09 by Linjudge FluA/pdm. RT-PCR revealed that one of these cases was H3N2 virus infection. Thus, the sensitivity of Linjudge FluA/pdm for H1N1pdm was 97.6% (41/42, 95%CI: 87.4–99.9).

**Table 4:** Sensitivity and specificity of Linjudge FluA/pdm.

Influenza A	Positive	Negative	TP^a^	FP^b^	TN^c^	FN^d^	% Sensitivity	95%CI	% Specificity	95%CI	PPV^e^	95%CI	NPV^f^	95%CI
96^1)^	42	54	41	4	50*	1	97.6	87.4–99.9	92.6	82.1–97.9	91.1	78.8–97.5	98	89.6–1.00
90^2)^	42	48	41	4	44	1	97.6	87.4–99.9	91.7	80–97.7	91.1	78.8–97.5	97.8	88.2–99.9

^a,^ true positive;

^b,^ false positive;

^c,^ true negative;

^d,^ false negative;

^e,^ positive predictive value;

^f,^ negative predictive value.

^1)^ influenza A by ImunoAce Flu;

^2)^, influenza A comfirmed by RT-PCR.

In contrast, among the 90 patients who were found to be positive for influenza A by ImunoAce Flu, 44 patients were found to be negative by Linjudge FluA/pdm and were confirmed to have A/H3N2 infection by RT-PCR. Moreover, 4 patients who were found to be positive by Linjudge FluA/pdm were diagnosed with H3N2 by RT-PCR. In total, 48 patients, including 4 patients for whom Linjudge FluA/pdm yielded a false-positive for H1N1pdm09, were diagnosed with H3N2 virus infection by RT-PCR. Thus, the specificity of Linjudge FluA/pdm was 91.7% (44/48, 95%CI: 80.0–97.7).

Moreover, 6 cases in which ImunoAce Flu yielded a false-positive result were found to be negative by Linjudge FluA/pdm. Thus, if the 6 cases were included in the analysis, the specificity of Linjudge FluA/pdm would be 92.6% (50/54, 95%CI: 82.1–97.9).

## Discussion

Based on a meta-analysis, RIDTs (*i*.*e*., antigen detection tests based on the immunochromatography) were reported to show low sensitivity (42.6%; 95%CI: 34.8–50.9) for influenza A in adult patients [9]. In contrast, an RIDT used in Japan, ImunoAce Flu, showed much 97.1% sensitivity in the detection of influenza A viruses (95%CI: 93.8–98.9) in adult patients (Table 1). The performance of RIDTs used in Japan might be higher than those reported in the meta-analysis [9]. In the same meta-analysis, it was also reported that RIDTs that utilize analyzer devices showed higher sensitivity in the detection of influenza viral antigens than RIDTs without analyzer devices. For example, the BD Veritor System, classified as “a digital immunoassay” in the meta-analysis report [9], showed 83.0% sensitivity and 97.5% specificity, which was much higher in comparison to RIDTs without analyzer devices. Although the BD Veritor System is also used in Japan, this superiority is not recognized. The minimum viral titer required for a positive reaction for influenza A viruses using the BD Veritor System (10^3^−10^4^ TCID50 per 100 μl) was similar to that required by ImunoAce Flu [13].

The sensitivity of RIDT is dependent on the viral load in the upper respiratory tract [1]. However, the timing at which samples were collected was not described in the recent meta-analyses that reported the low sensitivity of RIDTs [9, 10]. The Clinical Practice Guidelines by the Infectious Diseases Society of America (IDSA) emphasize that clinicians should collect upper respiratory tract specimens as soon as possible, preferably within 4 days after the onset of symptoms [18]. Accordingly, we believe that the recent reports [9, 10] included the results of many patients tested with RIDT kits at 4–5 days after the onset of illness. In contrast, in our study, most patients (89.8%) visited the outpatient department of our hospital within 48 hours after the onset of illness. Infectious influenza virus levels in the upper respiratory tract of persons with uncomplicated influenza peak during the first 1–2 days after illness onset, and decline to undetectable levels within a week [18]. In Japan, the early diagnosis and treatment of influenza are now standard practice [2].

Another important factor related to the high sensitivity of RIDTs in Japan was the measure used to collect samples for RIDTs. In our study, nasopharyngeal swabs were used as samples for all tests. In contrast, in the meta-analysis report, nasopharyngeal swabs or aspirate were used as test samples for <44% of tests [9]. The IDSA guidelines emphasize that nasopharyngeal specimens should be collected rather than other upper respiratory tract specimens in order to increase the detection of influenza [18].

ImunoAce Flu showed 89.2% specificity (95%CI: 84.1–93.1), which is relatively low (Table 1). When the study population was limited to patients who were tested within 48 hours after the onset of illness, the specificity was 90.4% (95%CI: 85.1–94.3) (Table 2). In contrast, the specificity was >99.9% (95%CI: 99.4–100) in the meta-analysis report [9]. The manuals for the other RIDT kits used in Japan report their sensitivity (88%–100%) and specificity (94%–100%) [19]. The low specificity in the present study may be attributable to the characteristics of the monoclonal antibody used in the ImunoAce Flu test kit.

The performance of RIDTs is usually discussed based on the clinical data in children. The performance is significantly better in children [20, 21], because age is inversely associated with viral load. This explains the better test results in children, and leads to some doubt regarding the diagnostic value of RIDTs in elderly persons [22]. In one meta-analysis report [10], the diagnostic test accuracy for influenza was significantly decreased in adults in comparison to children or a mixed population.

However, in our study, the sensitivity of ImunoAce Flu was sufficiently high in adults, especially in elderly individuals of >65 years of age (Table 1). Another Japanese report showed that the sensitivity did not differ between children and adults [23]. In that report, the sensitivity of a Japanese RIDT, Prolast Flu AB (Mitsubishi Chemical Medience Corporation, Japan) for H1N1pdm09 was as high as 80–90% during the H1N1 pandemic of 2009. In contrast, RIDTs were reported to show low sensitivity in the detection of influenza A/H1N1pdm09 virus (40–69%) in the H1N1 pandemic of 2009 in the US [24].

The IDSA guidelines recommend that clinicians use rapid molecular assays, that is, nucleic acid amplification tests (NAAT), rather than RIDTs for the testing of outpatients in order to improve the detection of influenza virus infection [18]. According to the meta-analysis [9], the pooled sensitivity and specificity of rapid molecular tests for influenza A were 87.4% (95%CI: 71.1–95.6) and 99.0% (95%CI: 93.2–99.5), respectively, in adults. The sensitivity and specificity of a rapid molecular test, ID NOW2 (Abott Diagnostics, USA), for influenza A were reported to be 95.9% (95%CI: 89.9–98.9) and of 100% (95%CI: 98.1–100), respectively [25], which was reported from Japan, and most patients—mainly children—were tested within 48 hours after the onset of illness. In our study, in which patients were tested within 48 hours after the onset of ILI, the sensitivity of ImunoAce Flu in the detection of influenza A was as high as the sensitivity of rapid molecular tests [25]. However, the specificity of ImunoAce Flu within 48 hours after the onset of illness was 90.4% (Table 2), which was lower than the specificity of rapid molecular assays [9] [25]. Similarly, in a report from Japan [25], sensitivity and specificity of an RIDT, QUICKNAVI (Otsuka Pharmaceutical Co, Japan) were comparable to the sensitivity and specificity of a rapid molecular assay, ID NOW2. Based on the Japanese experience, RIDTs are sufficiently sensitive and highly useful if patients are tested within 48 hours after the onset of illness. Thus, in countries where an early diagnosis and treatment are possible, an RIDT is probably one of the most useful alternatives for diagnosing influenza. For example, in Israel, >50% of patients with ILI visited clinics at 0–1 days after the onset of ILI [26].

The distinction of influenza A subtypes, A/H1N1pdm and A/H3N2, has been taken seriously because of characteristic clinical manifestations. Recently, however, it has become more important, mainly because of the lower effectiveness of the influenza vaccine against A/H3N2 [27, 28]. Moreover, resistance to a polymerase inhibitor, baloxavir marboxil, is frequently reported, especially in patients with influenza A/H3N2 [4, 29]. We could not distinguish between the influenza A subtypes by traditional RIDTs or rapid molecular assays, unless we used RT-PCR. However, with the combination of ImunoAce Flu and Linjudge FluA/pdm, distinction between influenza A subtypes becomes feasible.

Our study demonstrated the high sensitivity (97.6%; 95%CI: 87.4–99.9) and specificity (92.6%; 95%CI: 82.1–97.9) of Linjudge FluA/pdm in the detection of A/H1N1pdm09 (Table 4). Thus, by testing patients consecutively with ImunoAce Flu then Linjudge FluA/pdm, we are able to diagnose whether patients had A/H1N1pdm09 infection or A/H3M2 infection within a short time. ImunoAce Flu takes up to 5 minutes to obtain results [14], while Linjudge FluA/pdm takes up to 15 minutes [15]; however, highly accurate results are usually obtained within a total of 10 minutes. Another RIDT kit that is capable of distinguishing between the influenza A subtypes had been on the market until several years ago, Clearline Influenza A/B/(H1N1) 2009 [30]; however, it has since been withdrawn due to unstable results. The combination of ImunoAce Flu and Linjudge FluA/pdm is highly useful for distinguishing between influenza A subtypes, in comparison to RT-PCR, although the sensitivity and specificity of the combination of tests is lower. Moreover, this combination of tests obtains results in much less time, and numerous samples can be tested at the same time. The most important point is its cost, which at approximately 10 US dollars per test, is much cheaper in comparison to RT-PCR.

The present study was associated with several limitations. In some severe influenza cases, including cases with viral pneumonia [31], the infectious titer in the upper respiratory tract is reported to be low. Thus, negative test results in such cases should probably be confirmed by further testing using RT-PCR or other molecular assays to improve the detection of influenza virus infection [18]. In the present study, we only analyzed the performance of ImunoAce Flu in the detection of influenza A virus infection.

In conclusion, based on the Japanese experience, RIDTs are sufficiently sensitive and highly useful for the detection of influenza A if patients are tested within 48 hours after the onset of illness.
